# The efficiency and safety of multidetector computed tomography‐guided transseptal puncture during atrial fibrillation catheter ablation

**DOI:** 10.1002/joa3.12975

**Published:** 2023-12-12

**Authors:** Yi Lu, Zhen Yuan, Chunhui Liu, Shenghui Ma, Li Shu, Zhejun Cai

**Affiliations:** ^1^ Department of Cardiology Second Affiliated Hospital of Zhejiang University School of Medicine, Cardiovascular Key Lab of Zhejiang Province Hangzhou Zhejiang China

**Keywords:** atrial fibrillation, cardiac computed tomography, interatrial septum, intracardiac echo, transseptal puncture

## Abstract

**Background:**

Transseptal puncture (TSP) is a crucial technique for catheter ablation of atrial fibrillation (AF). Although intracardiac echo (ICE) facilitates a safe and accurate TSP, it is not widely used in developing countries because of the expense. This study evaluated the efficiency and safety of a novel cardiac multidetector computed tomography (MDCT)‐guided TSP during AF catheter ablation.

**Methods:**

The study consisted of two cohorts. In the index cohort, TSP procedure was performed under the guidance of ICE, and we recorded the angulation of right anterior oblique of X‐ray projection. In the validation cohort, we compared the efficiency and safety of TSP guided by MDCT‐calculated angulation with propensity‐score‐matched patients who underwent TSP guided by ICE.

**Results:**

We included 50 patients in the index cohort, and the mean angles of interatrial septum (IAS) measured from MDCT and ICE were 34.8 ± 6.3 and 35.1 ± 6.5, respectively. In the validation cohort, 376 patients were enrolled in the MDCT‐guided group and ICE‐guided group. Both groups had 1 case of cardiac tamponade. The mean axial plane angle was 35.46 ± 6.17 degrees, which was not influenced by age, gender, BMI, and LA size, while a moderate positive linear correlation between EF and the axial plane angle (*R* ^2^ = 0.14, *p* = .006).

**Conclusion:**

Cardiac MDCT can provide a clear vision of IAS orientation, and provide the appropriate RAO angle and height for TSP. The efficiency and safety of our MDCT‐guided TSP were comparable to ICE‐guided TSP, which may serve as an alternative method for TSP with ICE unavailable.

## Graphical abstract

Cardiac MDCT can provide a clear vision of IAS orientation, and provide the appropriate RAO angle and height for TSP. MDCT‐guided TSP may serve as an alternative method for TSP when ICE unavailable.

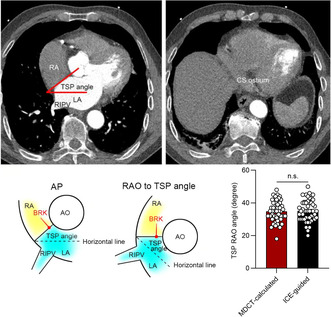

## 1 INTRODUCTION

Atrial fibrillation (AF) is the most common heart arrhythmia with increasing prevalence worldwide. It is associated with substantial morbidity and mortality, portending a heavy burden to patients and societal health.^1^ Catheter ablation is a well‐established therapy to restore and maintain sinus rhythm for AF patients. Pulmonary vein isolation is the cornerstone for AF catheter ablation. In order to access the left atrium through the systemic venous system and atrial septum, the transseptal puncture (TSP) technique was developed and refined in 1960s.^2^ Nowadays, TSP is a fundamental skill of cardiac electrophysiologist. In traditional practice, after withdrawing the sheath and needle into the fossa ovalis (FO), the exact puncture site is usually confirmed at 40° in right anterior oblique (RAO). In this angulation, the sheath rotated clockwise, making the tip of the sheath pointing to 12–1 o'clock, which runs posterior and parallel to the coronary sinus catheter. Then the puncture is preceded in the LAO projection.^3^ The insertion of needle into the left atrium is further confirmed by injecting contrast in a “straight line” manner.

However, the orientation of the interatrial septum (IAS) varies widely, the empirical practice could result in risky puncture and cardiac tamponade, even conducted by the most experienced electrophysiologist. Despite the developing techniques and tools for performing TSP, there is still 1% of complications associated with TSP, including life‐threatening cardiac tamponade and aortic root puncture.^4^ Intracardiac echo (ICE) permits visualization of the surrounding structures during TSP, which facilitates a safe and accurate TSP. However, because of the expense of ICE, it is not wildely used in developing countries.^5^

Contrast‐enhanced cardiac multidetector computed tomography (MDCT) is routinely performed before AF ablation to screen for potential left atrial appendage thrombosis. The morphology and orientation of the IAS can be clearly exhibited in cardiac MDCT scan. In this study, we sought to evaluate the efficiency and safety of MDCT‐guided TSP during AF catheter ablation.

## 2 MATERIALS AND METHODS

### 2.1 Study design

This is a retrospective study conducted among patients who underwent catheter ablation of AF between Jan 2021 and Apr 2022, in the Second Affiliated Hospital Zhejiang University School of Medicine. The main inclusion criteria were (1) diagnosed as atrial fibrillation with failed anti‐arrhythmic drug therapy; (2) glomerular filtration rate >60 mL/min measured using the CKD‐EPI equation and without an allergic history of iodic contrast medium; and (3) obtained written informed consent. The main exclusion criteria were (1) left atrial appendage thrombosis and (2) contraindicate to oral anticoagulants.

For the present study, we included 2 cohorts of patients. The index cohort included 50 patients. TSP procedure was performed under the guidance of ICE, and the angulation of RAO of X‐ray projection was recorded and compared with the angulation in MDCT scan. The validation cohort consisted of 376 patients who underwent TSP guided by MDCT‐calculated angulation. Another 376 propensity‐score‐matched patients who underwent TSP guided by ICE were also included in the validation cohort.

The primary endpoints were TSP‐related complication, including cardiac tamponade, aortic root puncture, and peri‐operative death. The study was conducted on the grounds of the Declaration of Helsinki and approved by the Ethics Committee of the Second Affiliated Hospital, Zhejiang University School of Medicine.

### 2.2 Measurement of TSP angulation in MDCT

Contrast‐enhanced cardiac MDCT was obtained 1–3 days prior to AF ablation, which was performed using a 128‐detector MDCT (Siemens SOMATOM definition flash CT).

**FIGURE 1:**
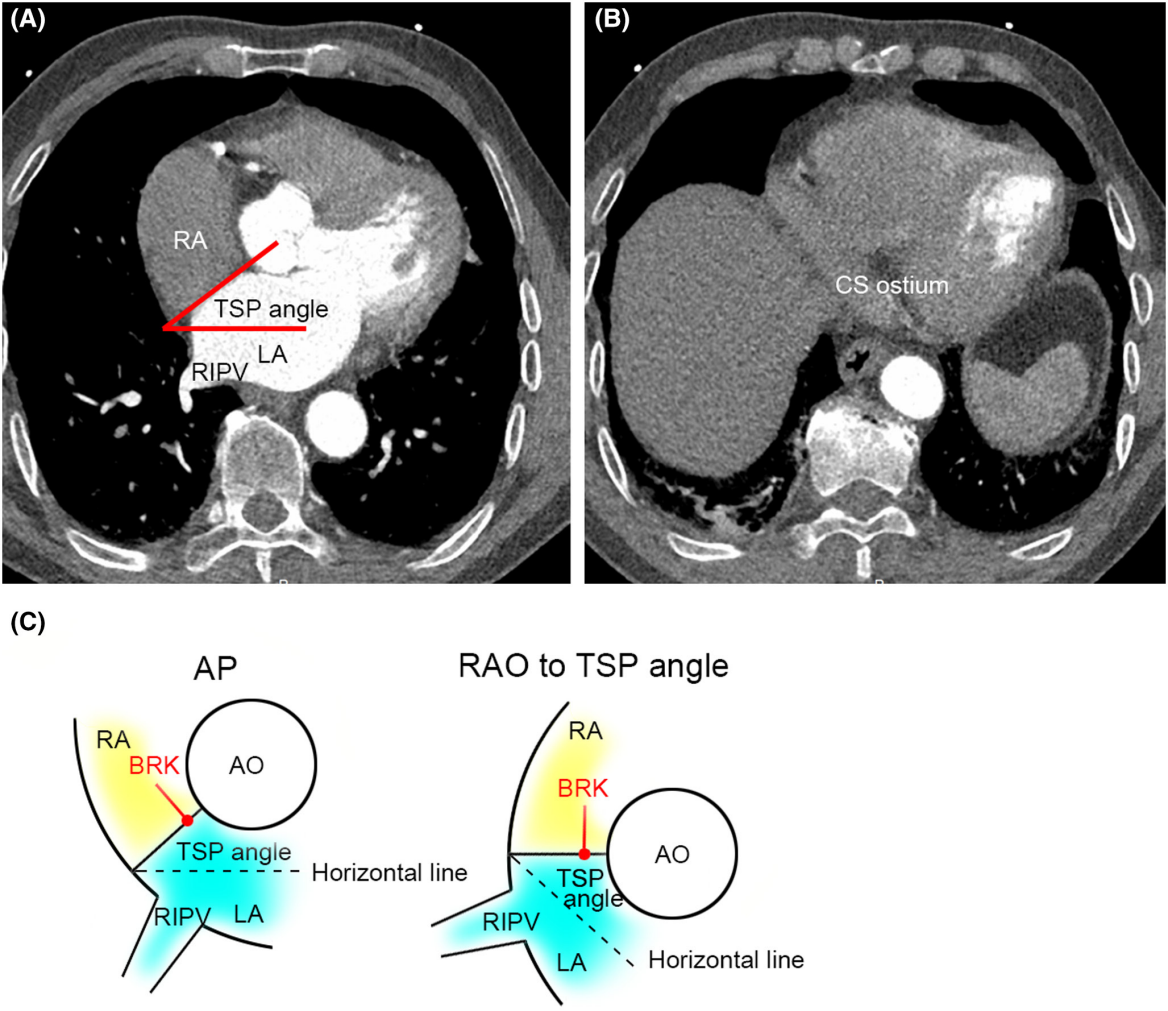
An example and schema of interatrial septum angle measurement. (A) The plane view of right inferior pulmonary vein was utilized to calculate the angulation. (B) An example of identification of coronary sinus ostium in the multidetector computed tomography. Note the low CT value area between left atrium and ventricle. (C) The schema of image measurement from the CT scan in anteroposterior view and right anterior oblique view during the TSP practice. By rotating the C‐arm to right anterior oblique at a certain angle measured from the left panel, the tip of needle is perpendicular to the fossa ovalis. AO, aorta; AP, anteroposterior; BRK, Brockenbrough needle; LA, left atrium; RA, right atrium; RAO, right anterior oblique; RIPV, right inferior pulmonary vein; TSP, transseptal puncture.

The IAS planes at the FO and bottom of coronary sinus (CS) ostium were identified in atrial end‐systolic axial images by 2 dependent researchers. The plane view of right inferior pulmonary vein was utilized to calculate the angulation. The angle of IAS and horizontal line was determined as the best RAO angulation for TSP. The angulation was calculated as the mean value of 2 results. The sagittal location for TSP was determined by the distance between the bottom of CS ostium and FO, by multiplying the scan slice thickness and sections between CS and FO. An example of image measurement is provided in Figure 1.

### 2.3 Details of the transseptal puncture procedure

The procedure of TSP and catheter ablation was performed by three experienced electrophysiologists in our center. Before TSP, the decapoloar catheters were introduced into CS via the inferior vena cava approach, which indicated the bottom of CS ostium. Standard adult 71 cm Brockenbrough (BRK) needle (St Jude Medical, USA) and 8.5F Swartz SL1 sheath (St Jude Medical, USA) were used for TSP. The SL1 sheath was advanced through a J‐tipped guidewire into the superior vena cava (SVC) via femoral vein, and the guidewire was exchanged for the BRK needle. The SL1 and BRK needle were both rotated to approximately 4–5 o'clock position as described before,^3^ and withdrew from the SVC slowly and observed two falls in the anteroposterior fluoroscopic view. The second fall indicated the landing of needle tip into the FO. The sagittal location for TSP was measured from MDCT ahead of the procedure as described before. A good landing zone was defined as reconfirmed in the X‐ray by measuring the distance between BRK needle's tip and the decapolar catheter in CS.

**FIGURE 2:**
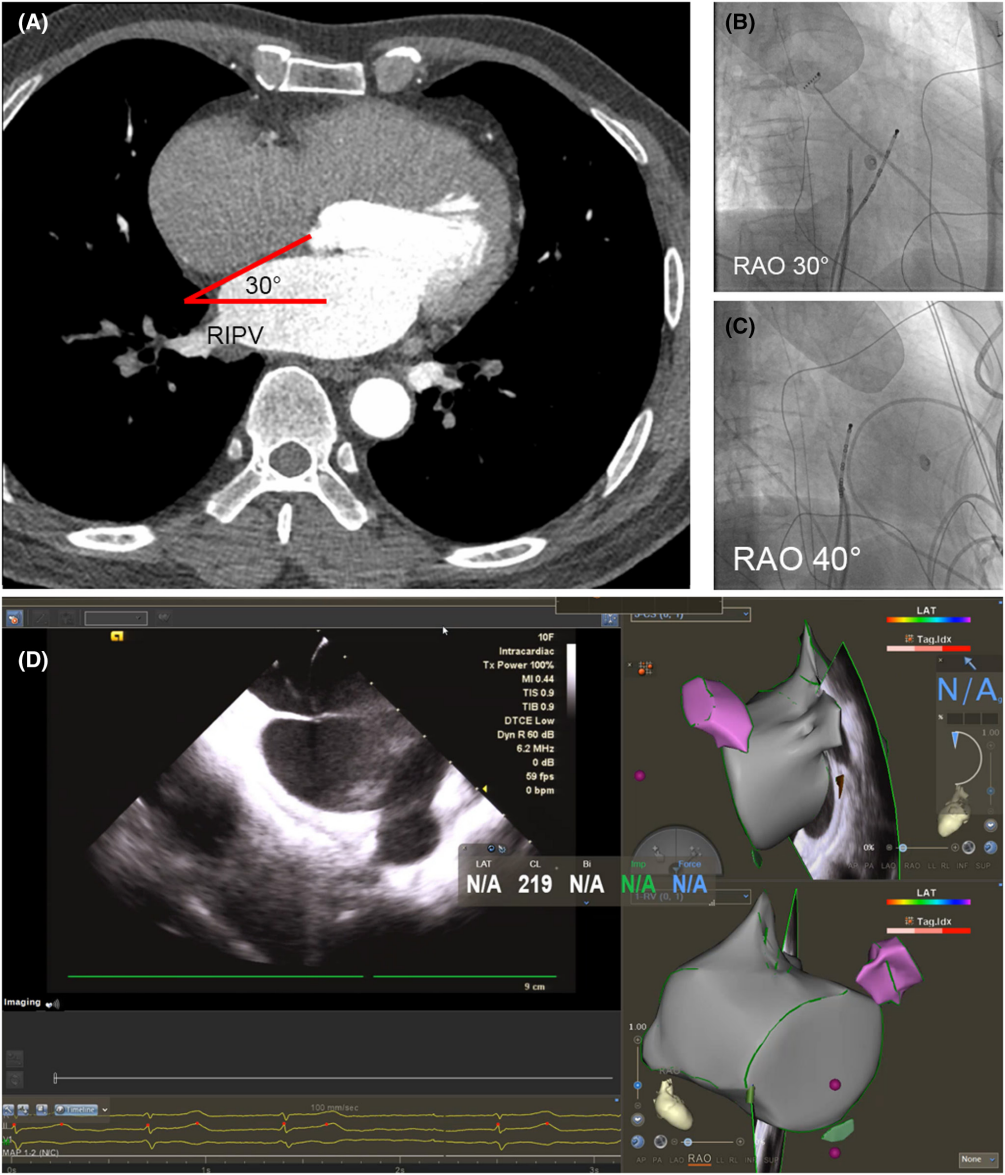
An example of transseptal puncture guided by multidetector computed tomography (MDCT) and verified by intracardiac echo in the index cohort. (A) The orientation of IAS was 30° measured from MDCT scan. (B, C) The tip of sheath pointed to 12 o'clock in the X‐ray image from RAO 30°, while pointed to 11 o'clock in traditional RAO 40°. (D) Intracardiac echo visualized the left upper pulmonary vein and fossa ovalis (FO), with “tenting” within the middle of the FO.

In the index cohort, ICE was introduced into the right atrium via a 11F sheath (St Jude Medical, USA), and rotated clockwise from home view, until it visualized the left upper pulmonary vein and FO in the constant view field. The “tenting” within the middle of the FO was defined as a suitable puncture site. Meanwhile, the C‐arm was rotated rightward until the tip of the sheath was pointing to the 12 o'clock in the X‐ray image, that is, in a straight line with the body of sheath. The angulation of RAO of X‐ray projection was recorded (Figure 2). Then the needle was advanced to the left atrium and arterial blood could be withdrawn from the needle. The location of the tip of the needle was confirmed by injecting saline, arousing bubbles within LA in ICE view. The puncture was then preceded as described before.^5^

In the validation cohort, 376 patients were enrolled in the MDCT‐guided group. The atrial septal angulation was measured from MDCT before TSP. During the puncture procedure, the calculated angle was applied in RAO view. The sheath was rotated clockwise until its tip pointed to the 12 o'clock in the X‐ray image. After the engagement of the FO, the needle was advanced to the LA and was confirmed at 45° in left anterior oblique (LAO) projection by injecting contrast following a straight line pattern.

Meanwhile, another 592 AF patients underwent ICE‐guided TSP and catheter ablation in our center. We conducted 1:1 propensity score matching (PSM) and enrolled another 376 patients in the control group.

### 2.4 Statistical analyses

Data were presented as means ± standard deviation (SD) or median (25th and 75th percentiles) for continuous variables, as appropriate. Data were expressed as the number (percentage) for qualitative variables. The chi‐squared test was used to compare categorical variables, and Student's *t*‐test or the Mann–Whitney test was used for continuous variables between the two groups.

Age, gender, BMI, CHADS2‐VASc score, HAS‐BLED score, LA diameter, and EF were utilized to perform 1:1 PSM with a multivariable logistic regression model. We performed PSM using the nearest neighbor matching, with a default caliper of 0.1. The association between the axial plane angle and age, gender, BMI, LA size, and EF was analyzed by univariate linear regression. All statistical analyses were performed using IBM SPSS Statistics for Windows, version 26.0 (IBM, Armonk, New York).

## 3 RESULTS

### 3.1 Comparison of MDCT‐calculated and ICE‐guided TSP angles

**FIGURE 3:**
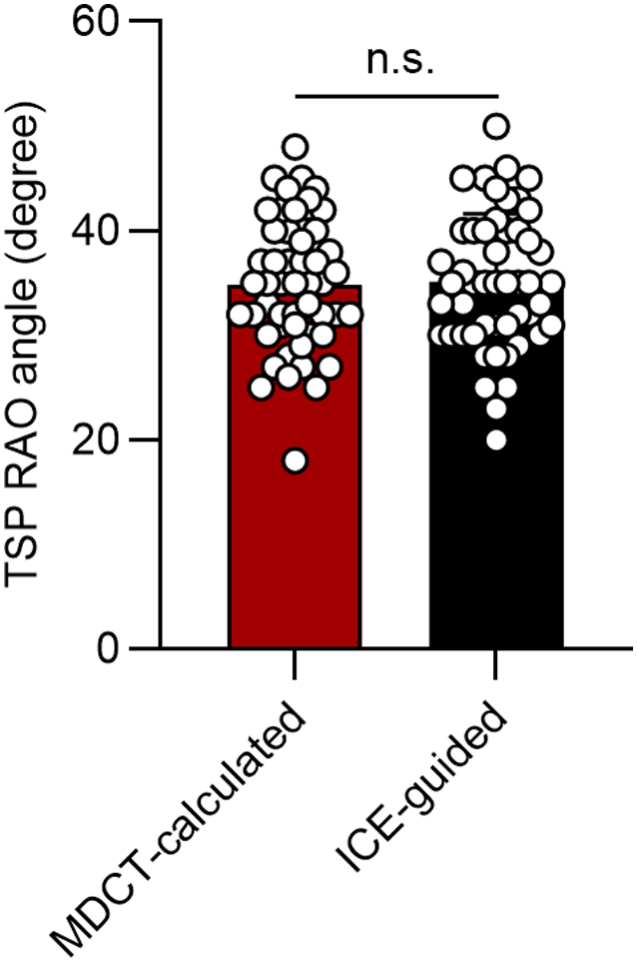
Comparison of TSP RAO angles measured by multidetector computed tomography and intracardiac echo. The mean angles measured from multidetector computed tomography and intracardiac echo were 34.8 ± 6.3 and 35.1 ± 6.5, and there was no difference between the two groups. MDCT, multidetector computed tomography; RAO, right anterior oblique; TSP, transseptal puncture.

We first included 50 patients in the index cohort, and compared the MDCT‐calculated TSP angle with ICE‐guided TSP angle. The mean age of patients was 63.4 ± 9.5 years old and 31/50 were male. The mean angles measured from MDCT and ICE were 34.8 ± 6.3 and 35.1 ± 6.5, respectively (Figure 3). Paired student's *t*‐tests found no significant difference between the two methods (*p* = .103).

### 3.2 Validation of MDCT‐guided TSP

Since the data in the index cohort showed comparable results between MDCT‐calculated and ICE‐guided TSP angles, we next sought to validate whether our MDCT‐guided method is feasible in a larger cohort. In total, 376 patients were enrolled in the MDCT‐guided group. Meanwhile, 592 patients with atrial fibrillation underwent ICE‐guided TSP. We conducted 1:1 propensity score matching with age, gender, BMI, CHADS2‐VASc score, HAS‐BLED score, LA diameter, and EF, yielding a comparison group named as ICE‐guided. Demographic and clinical characteristics of MDCT‐guided and ICE‐guided TSP groups were presented in Table 1. We also investigated the relevant factors that might infer with our TSP procedure, including a history of cardiac surgery, pacemaker implantation, and previous history of AF catheter ablation. Still, there was no significant difference between the 2 groups.

**TABLE 1:** Demographic and clinical characteristics of patients in baseline.

Variables	ICE‐guided (*n* = 376)	MDCT‐guided (*n* = 376)	*p* value
Age	63.97 ± 9.33	63.79 ± 10.70	.811
BMI	24.67 ± 3.06	24.68 ± 3.94	.979
Male gender	227 (60.37%)	235 (62.50%)	.599
CHADS2‐VASc	2.25 ± 1.57	2.17 ± 1.59	.518
HAS‐BLED	1.45 ± 1.12	1.37 ± 1.03	.313
LA size (cm)	4.17 ± 0.64	4.08 ± 0.64	.053
EF (%)	62.46 ± 9.97	62.34 ± 10.62	.855

Abbreviations: BMI, body mass index; EF, ejection fraction; ICE, intracardiac echo; LA size, left atrium size; MDCT, multidetector computed tomography.

All patients underwent catheter ablation successfully, except one case of cardiac tamponade in each group (Table 2). Patients with cardiac tamponade underwent urgent pericardiocentesis and drainage and recovered smoothly afterward. However, since both cases of cardiac tamponade occurred after the start of catheter ablation and were relieved after pericardial drainage, it was difficult to distinguish the cause of tamponade between misconduct of TSP or ablation.

**TABLE 2:** Complications during procedures.

	ICE‐guided (*n* = 376)	MDCT‐guided (*n* = 376)	*p* value
Success rate	100%	100%	.997
Cardiac tamponade	1	1	.997
Aortic root rupture	0	0	.997

Abbreviations: ICE, intracardiac echo; MDCT, multidetector computed tomography.

Notably, the septal orientation in the axial plane varied widely, ranging from 16° to 53°. The mean axial plane angle was 35.46 ± 6.17 degrees. We further studied the association of IAS angle with age, BMI, gender, LA size, and EF. The results showed that the axial plane angle was not influenced by age, gender, BMI, and LA size, while a moderate positive linear correlation was observed between EF and the axial plane angle (*R* ^2^ = 0.14, *p* = .006).

## 4 DISCUSSION

TSP could result in life‐threatening complications and was traditionally conducted at 40° in RAO projection. Previous study showed that the best angle for projecting the septum was RAO 36°.^6^ However, according to the present study, the orientation of the IAS varies widely, ranging from 16° to 53°, which poses challenges to the traditional practice of TSP with fixed RAO X‐ray projection. More importantly, this angle was not influenced by age, gender, BMI, and LA size. In other words, the IAS angulation is not predictable.

ICE improves the safety of TSP especially in challenging cardiac anatomy (including thick, aneurysmal, and/or excessively mobile septum, and patients), the detailed anatomy and livetime view of ICE lead to significantly increased safety for TSP. Also, in cases with excessive septal tenting, which poses a risk of cardiac perforation, ICE imaging may be safer.^7^ However, it increases the expense and procedural time of AF catheter ablation significantly. Extra venous access and the relatively large diameter of ICE also give rise to the likelihood of local puncture injury. Besides, the expense of ICE may also limit the use in developing countries.

Cardiac MDCT can exhibit a clear vision of IAS, providing information like atrial septal aneurysm, thickness of IAS and IAS orientation, and may assist the process of TSP. In this study, we reported a novel and easy way to adjust the projection of X‐ray during TSP procedural according to the IAS orientation measured from the MDCT scan. There was also other MDCT‐guided method reported previously.^6^ However, with only one plane to determine the angulation, our method is more straightforward.

## 5 CONCLUSIONS

The efficiency and safety of our MDCT‐guided TSP were comparable to ICE‐guided TSP, which may serve as an alternative method for TSP with ICE unavailable.

### 5.1 Study limitations

This study has several limitations. First, this study was not a randomized study. Despite the fact that we conducted PSM, it might still miss important influencers for TSP. Second, in the MDCT‐guided group, the TSP was preceded when the sheath's tip pointed to the 12 o'clock in the X‐ray image. The exact direction might be influenced by the judgment of electrophysiologists.

## AUTHOR CONTRIBUTIONS

Zhejun Cai and Yi Lu designed the study. Yi Lu, Zhejun Cai, and Shenghui Ma conducted the TSP procedural and collected data. Yi Lu and Zhen Yuan drafted the manuscript. All authors were involved in critically revising the manuscript.

## CONFLICT OF INTEREST STATEMENT

Authors declare no conflict of interests for this article.

## ETHICS STATEMENT

The study was approved by the Ethics Committee of the Second Affiliated Hospital, Zhejiang University School of Medicine.

## PATIENT CONSENT STATEMENT

All patients signed the informed consent.

## Data Availability

The data that support the findings of this study are available from the corresponding author upon reasonable request.

## References

[1] Hindricks G , Potpara T , Dagres N , Arbelo E , Bax JJ , Blomström‐Lundqvist C , et al. 2020 ESC Guidelines for the diagnosis and management of atrial fibrillation developed in collaboration with the European Association for Cardio‐Thoracic Surgery (EACTS): the task force for the diagnosis and management of atrial fibrillation of the European Society of Cardiology (ESC) developed with the special contribution of the European Heart Rhythm Association (EHRA) of the ESC. Eur Heart J. 2021;42:373–498. 32860505 10.1093/eurheartj/ehaa612

[2] Brockenbrough EC , Braunwald E , Ross J Jr . Transseptal left heart catheterization. A review of 450 studies and description of an improved technic. Circulation. 1962;25:15–21. 13873261 10.1161/01.cir.25.1.15

[3] Earley MJ . How to perform a transseptal puncture. Heart. 2009;95:85–92. 19047447 10.1136/hrt.2007.135939

[4] Katritsis GD , Siontis GC , Giazitzoglou E , Fragakis N , Katritsis DG . Complications of transseptal catheterization for different cardiac procedures. Int J Cardiol. 2013;168:5352–5354. 24012276 10.1016/j.ijcard.2013.08.004

[5] Ruisi CP , Brysiewicz N , Asnes JD , Sugeng L , Marieb M , Clancy J , et al. Use of intracardiac echocardiography during atrial fibrillation ablation. Pacing Clin Electrophysiol. 2013;36:781–788. 23305194 10.1111/pace.12030

[6] Wang Y , Chen G , Bai Y , Li S , Natale A , Dong J , et al. Transseptal puncture by CTP‐2 method: results from cardiac computed tomography analysis and clinical application. Medicine (Baltimore). 2016;95:e4504. 27559952 10.1097/MD.0000000000004504 PMC5400318

[7] Manolis AS . Transseptal access to the left atrium: tips and tricks to keep it safe derived from single operator experience and review of the literature. Curr Cardiol Rev. 2017;13:305–318. 28969539 10.2174/1573403X13666170927122036 PMC5730964

